# Serum Interleukin-37 Increases in Patients after Ischemic Stroke and Is Associated with Stroke Recurrence

**DOI:** 10.1155/2021/5546991

**Published:** 2021-04-13

**Authors:** Ying Zhang, Chengbi Xu, Haitao Wang, Shanji Nan

**Affiliations:** ^1^Department of Neurology, Second Hospital of Jilin University, Changchun, China; ^2^Department of Otolaryngology, Second Hospital of Jilin University, Changchun, China

## Abstract

**Background:**

This study seeks to assess interleukin-37 (IL-37) serum level in acute ischemic stroke and the value of predicting 3-month stroke recurrence and functional outcome in acute ischemic stroke.

**Methods:**

From January 1, 2018, to June 30, 2019, all consecutive first-ever acute ischemic stroke patients from our hospital, China, were included. Serum samples, clinical information, and stroke severity (defined by the National Institute of Health stroke scale (NIHSS) score) were collected at baseline. Serum IL-37 level was measured by the enzyme-linked immunosorbent assay (ELISA) method. Functional impairment (defined by the modified Rankin scale (mRS)) and recurrent stroke were assessed 3 months after admission. The relation of IL-37 with either clinical severity at baseline, unfavorable functional outcome, or stroke recurrence at follow-up was evaluated by logistic regression analysis, and the results were presented as odds ratios (OR) with 95% confidence intervals (CI).

**Results:**

Three hundred and ten stroke patients were included. The median IL-37 serum level in those patients was 344.1 pg/ml (interquartile range (IQR), 284.4-405.3 vs. control cases: 122.3 pg/ml (IQR, 104.4-1444.0); *P* < 0.001). At 3 months, a total of 36 (11.6%) patients had a stroke recurrence. IL-37 serum levels in those patients were higher than in those patients without stroke recurrence (417.0 pg/ml (IQR, 359.3-436.1) vs. 333.3 pg/ml (279.0-391.0)). In a logistic model adjusted for other factors, IL-37 in the highest quartile (>405.3 pg/ml) was still associated with recurrent stroke (OR = 3.32; 95%CI = 2.03–6.13; *P* < 0.001). IL-37 could promote the NIHSS score (area under the curve (AUC) of the IL-37/NIHSS, 0.75; 95% CI, 0.67–0.83; *P* < 0.001), corresponding to a difference of 0.085 (0.005). Serum IL-37 increases in patients with poor outcome, and an IL-37 in the highest quartile is related to poor outcome (OR = 4.85; 95%CI = 3.11 − 8.22; *P* < 0.001).

**Conclusion:**

Serum IL-37 increased in patients after ischemic stroke and was associated with stroke recurrence events and poor stroke outcomes. Large randomized controlled trials should be carried out to confirm whether IL-37 lowering treatment improves stroke prognosis.

## 1. Introduction

Stroke is one of the leading causes of mortality and long-term disability in China [1, 2]. It has become a public health problem and adds a heavy medical burden to our country [3]. Wu et al. [4] suggested that stroke was associated with the highest disability-adjusted life-years lost and 2 million new cases per year. The proportion of the population aged 65 and above in China will be tripled from 9.6% in 2015 to 27.6% in 2050 [5]. As an aging disease, stroke prevalence is expected to increase significantly in the next ten years [6].

Recurrent stroke is a frequent complication after stroke. Han et al. [7] showed that the overall age-standardized rate of recurrent stroke within one year and five years was 5.7% and 22.5%, respectively. The recurrent stroke could greatly promote the risk of disability and death [7]. One study indicated that stroke patients with recurrence events impacted 3 months of unfavorable functional outcomes [8], and another study confirmed that recurrent stroke had been associated with increased mortality and functional dependence [9]. Therefore, proactive early prediction of stroke recurrence is of great significance for improving the prognosis of patients.

Many of the biomarkers identified relate to ischemic stroke's pathophysiology, including diagnosis, prediction of stroke severity, and outcome [10]. Inflammatory reactions and processes play an essential role in the pathophysiological process of ischemic stroke [11]. High levels of inflammatory biomarkers, such as C-reactive protein (CRP) or interleukin-6 (IL-6), were associated with an unfavorable functional outcome after stroke [12]. Nakase et al. [13] showed that IL-6 might predict both the severity of the stroke lesions and patients' outcomes. Furthermore, inflammatory markers are related to recurrent vascular events after stroke [14]. Castillo et al. [15] showed that inflammation markers are related to the risk of recurrence of vascular disease following a stroke. The evidence presented in another study suggested that elevated levels of CRP were associated with the risk of new cardiovascular events after ischemic stroke [16].

Interleukin-37 (IL-37), a member of the proinflammatory IL-1 superfamily, plays a role in inflammatory diseases [17], such as atherosclerosis [18], cardiovascular disease [19, 20], and stroke [21]. The potential mechanism role of IL-37 in the pathophysiological of stroke recurrence events after ischemic stroke is not apparent. This study sets out to investigate IL-37 serum level in acute ischemic stroke and the value of predicting 3-month stroke recurrence and functional outcome in acute ischemic stroke.

## 2. Methods

### 2.1. Patients

From January 1, 2018, to June 30, 2019, all consecutive first-ever acute ischemic stroke patients (within 48 hours of the onset of symptoms) from Second Hospital of Jilin University, China, were included. Acute ischemic stroke was diagnosed by the World Health Organization criteria [22] and confirmed by Magnetic Resonance Imaging (MRI) and/or Computed Tomography (CT) scans. The exclusion criteria of the study included malignant tumor, intracerebral hemorrhage, renal and liver insufficiency, serious infections, and autoimmune diseases. Those patients who lack informed consent, symptoms that appear for more than 48 hours, and lifeless than three months were also excluded.

One hundred and ten healthy participants (age and sex-matched) from the Health Physical Examination Center (HPEC) of our hospital were assigned as the normal control group. Those participants' median age was 66 years (interquartile ranges (IQR), 56-75), and 58.1% were male. All individuals with symptoms of cerebrovascular disease and the above exclusion criteria should be excluded.

### 2.2. Ethics

Our research protocol was approved by the ethics committee of Second Hospital of Jilin University, according to the 1964 Declaration of Helsinki. All participants should sign an informed consent before inclusion in the study. For some patients who were unconscious, family members' consent was also acceptable.

### 2.3. Clinical Variable Collection

We collected the following clinical information: demographic characteristics (age, sex, ethnicity, urban and rural residence, height, and weight), vascular risk factors (hypertension, diabetes mellitus, hypercholesterolemia, obesity (defined by body mass index (BMI) ≥ 28 kg/m^2^), atrial fibrillation (AF), a history of transient ischemic attack (TIA), and family history of stroke, smoking habit, and alcohol abuse), and prestroke treatment strategy (antihypertensive, hypoglycemic, anticoagulants, and antiplatelet). Also, acute treatment, including IV thrombolysis and/or mechanical thrombectomy, was also recorded.

On admission, stroke severity was analyzed by a stroke neurologist according to the National Institute of Health Stroke Scale (NIHSS; a score from 0 to 42, the higher the score, the more serious the stroke) [23] score. Stroke etiology was divided into five categories according to the TOAST (Trial of Org 10172 in Acute Stroke Treatment) classification: large-vessel occlusive, small-vessel occlusive, cardioembolic, other, and unknown [24]. Some patients performed MRI examination, and the infarct area was calculated using the formula 0.5 × *a* × *b* × *c* [25].

### 2.4. Follow-Up and Stroke Recurrence

All surviving patients would be followed up in an outpatient clinic at three months from baseline. A standard questionnaire was used. All patients received (MRI) and/or CT scans. Stroke recurrence events were checked and recorded (diagnostic criteria: ① a sudden functional deterioration (a decrease of the NIHSS ≥ 4); ② a new focal neurological deficit of vascular origin lasting > 24 hours) [26, 27]. Poor functional outcome was assessed using the modified Rankin Scale (mRS) [28] with a score of 3 to 6 points [29].

### 2.5. Laboratory Testing

Fasting serum samples were collected in the first morning (6 to 8 a.m.) after admission. Serum levels of IL-37 were tested by enzyme-linked immunosorbent assays (ELISA) (Cusabio Technology LLC, Wuhan, China). The detection limit and range of the standard curve for IL-37 were 7.8 pg/ml and 31.3 pg/mL-2000 pg/ml. Intra-assay and interassay precisions were 6.6% and 8.4%, respectively. Serum levels of glucose, CRP, and IL-6 were also tested by the standard test method.

### 2.6. Statistical Analysis

The data were divided into categorical and continuous variables, and the results were shown as number (percentage) and median (interquartile ranges (IQRs)). The chi-square test (categorical variables) and Mann–Whitney *U* test (continuous variables) were used to compare groups. Spearman's rank correlation was used to assess the correlation of the factors.

The relation of IL-37 with either clinical severity at baseline, unfavorable functional outcome, or stroke recurrence at follow-up was evaluated by logistic regression analysis, and the results were presented as odds ratios (OR) with 95% confidence intervals (CI). Clinical severity at baseline was dichotomized as high clinical severity (NIHSS ≥ 6) and low severity (NIHSS < 6) [30]. The reference used for the analyses was the lowest three quartiles (Q1-Q3), corresponding to an IL − 37 level ≤ 405.3 pg/ml (Q4 vs. Q1-Q3). The multivariable model included demographic characteristics, BMI, stroke subtype, vascular risk factors, prestroke and acute treatment strategies, and serum levels of glucose, IL-6, CRP, and IL-37.

The accuracy of IL-37 and other factors to predict stroke recurrence was analyzed by receiver operating characteristic (ROC) curve, and the results were presented as the area under the curve (AUC) with 95% CI. The accuracy of the combined model (NIHSS and IL-37) was compared with NIHSS only. Statistical significance was set at *P* < 0.05 (two-tailed). Statistical analysis was performed with SPSS 24.0 (SPSS Inc., Chicago, IL, USA) and the ROCR package (Version 1.0-2).

## 3. Results

### 3.1. Baseline Characteristics of the Study Population

**Figure 1:**
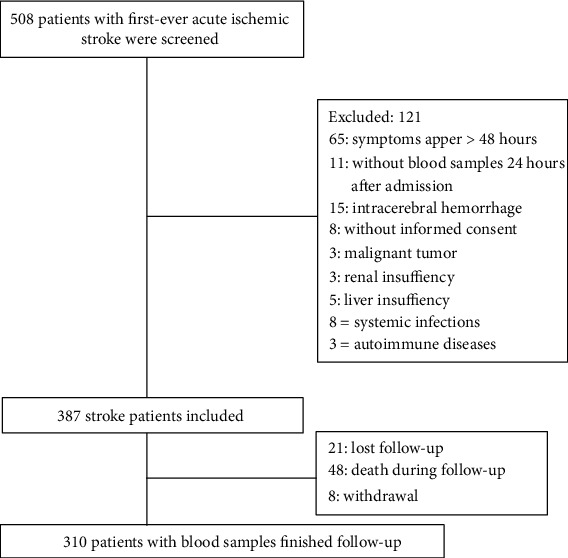
Study profile/flow sheet of the study.

As shown in Figure 1, a total of 508 eligible patients with acute ischemic stroke, 387 patients were included. Finally, 310 patients (the median age was 66 years (IQR, 56-75) and 48.2% were men) with blood samples finished follow-up (48 patients died during follow-up, 21 patients lost follow-up, and eight withdrawal). However, the baseline characteristics in those patients were compared to the overall cohort (age (*P* = 0.13), sex (*P* = 0.75), BMI (*P* = 0.22), and NIHSS (*P* = 0.09)). On admission, the median NIHSS score was 6 points (IQR, 3 to 11), and lesion volume (*N* = 175) was 15 ml (IQR, 5-31). More information were described in Table 1.

**Figure 2:**
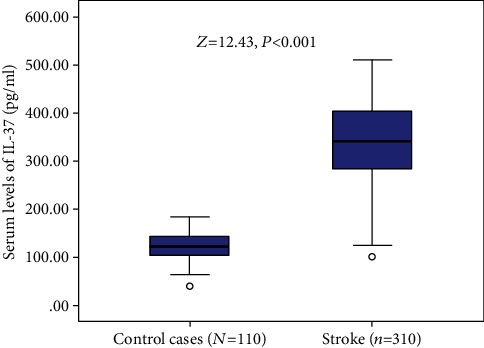
Serum levels of IL-37 in stroke patients and control cases. All data are medians and inter-quartile ranges (IQR). *P* values refer to Mann–Whitney *U* tests for differences between groups.

As shown in Figure 2, the median IL-37 serum level in those stroke patients was 344.1 pg/ml (IQR, 284.4-405.3), which was higher than in those control cases (122.3 pg/ml (IQR, 104.4-1444.0)). According to ROC analysis, the cut-off value of IL-37 to diagnose ischemic stroke was suggested to be 193.0 pg/ml, with the AUC (95% CI) of 0.98 (0.96-0.99). The sensitivity and specificity at this concentration were 95.2% and 97.3%, respectively.

### 3.2. Serum Levels of IL-37 and Stroke Characteristics

**Figure 3:**
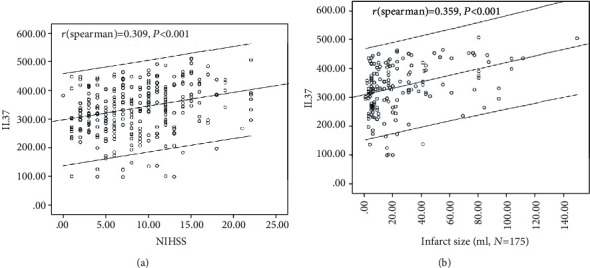
Correlation between serum IL-37 level and other factors. (a) Correlation between the NIHSS and serum IL-37 level; (b) correlation between the infarct area and serum IL-37 level.

The NIHSS and serum IL-37 levels were positively correlated (*r* = 0.309, *P* < 0.001) (Figure 3(a)). On admission, 141 patients (45.4%) were defined as minor stroke (NIHSS < 6). The IL-37 in those patients was lower (*P* = 0.001) than in those patients with moderate-to-high clinical severity (322.6 pg/ml (IQR, 269.5-366.8) vs. 364.4 pg/ml (292.4-417.0)). According to ROC analysis, the cut-off value of IL-37 to diagnose moderate-to-high clinical severity (NIHSS > 5) was suggested to be 374.0 pg/ml, with the AUC (95% CI) of 0.62 (0.56-0.68). The sensitivity and specificity at this concentration were 45.6% and 77.9%, respectively. In multivariate logistic regression analysis, IL − 37 > 405.3 pg/ml was associated with an increased risk of a moderate-to-high clinical severity (OR = 2.85; 95%CI = 2.14 − 3.76; *P* = 0.003).

In patients for whom MRI lesion volumes were available (*N* = 175), the lesion size and serum IL-37 levels were positively correlated (*r* = 0.359, *P* < 0.001), Figure 3(b). Interestingly, there was significantly positive relevance between levels of IL-37 and IL-6 (*P* = 0.001) and CRP (*P* = 0.018). Besides, the relevance between serum IL-37 levels and stroke subtypes was assessed. The IL-37 serum levels in cardioembolic stroke were greater than in those other subtypes (355.5 pg/ml (IQR, 285.4-410.3) vs. 329.3 pg/ml (IQR, 274.3-375.3) mg/L; *P* = 0.002).

### 3.3. IL-37 Serum Levels and 3-Month Stroke Recurrence

**Figure 4:**
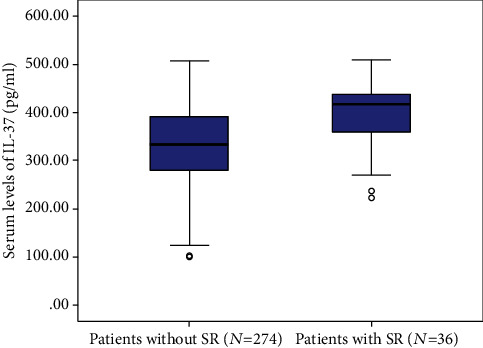
Serum levels of IL-37 in stroke patients with and without stroke recurrence. All data are medians and interquartile ranges (IQR). *P* values refer to Mann–Whitney *U* tests for differences between groups. SR: stroke recurrence.

At 3 months, a total of 36 patients (11.6%) had a stroke recurrence. As shown in Figure 4, IL-37 serum levels in those patients were higher than in those patients free of stroke recurrence (417.0 pg/ml (IQR, 359.3-436.1) vs. 333.3 pg/ml (279.0-391.0)). Univariate logistic regression models showed that an IL-37 in the highest quartile was associated with stroke recurrence (OR = 4.55; 95%CI = 2.17–9.13; *P* < 0.001). In a multivariate logistic model adjusted for other factors, an IL-37 in the highest quartile was still associated with stroke recurrence (OR = 3.32; 95%CI = 2.03–6.13; *P* < 0.001). NIHSS, stroke etiology, atrial fibrillation, acute treatment, serum levels of IL-6, and CRP remained significant recurrence predictors (Table 2). In those patients with MRI testing (*n* = 175), IL-37 in the highest quartile was also an independent recurrence predictor with an OR of 2.95 (95% CI, 1.87-6.02; *P* = 0.001) after adjustment for both NIHSS score and lesion volume.

**Figure 5:**
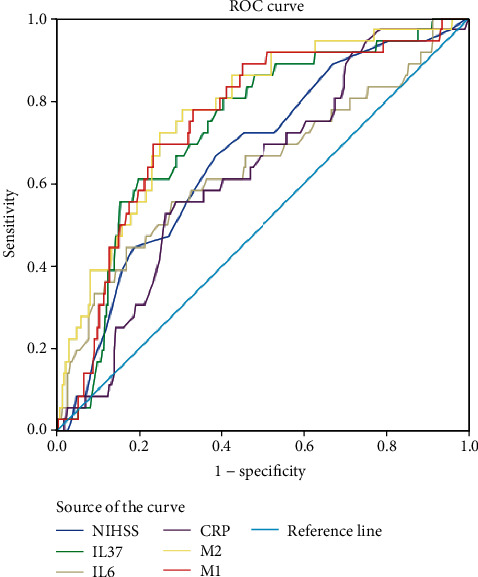
Receiver operator characteristic curve demonstrating sensitivity as a function of 1-specificity for predicting the functional outcome within 3 months based on the logistic model incorporating all 4 biomarkers/NHISS and the relative contribution of each biomarker alone (initial cohort). M1 included IL-37 and NHISS Score, M2 included IL-37, IL-6, and CRP. NIHSS: National Institutes of Health Stroke Scale; CRP: C-reactive protein; IL-37: interleukin-37; IL-6: interleukin-6.

According to ROC analysis, the cut-off value of IL-37 to predict recurrence was suggested to be 406.8 pg/ml, with the AUC (95% CI) of 0.73(0.65-0.82). The sensitivity and specificity at this concentration were 61.1% and 80.3%, respectively. As shown in Figure 5, a ROC curve analysis was used to compare the prognostic accuracy of the NIHSS, CRP, IL-6, and IL-37. The prognostic accuracy of IL-37 was high compared to that of NIHSS (AUC [95% CI]: 0.67 [0.58-0.76]), IL-6 (AUC [95% CI]: 0.64 [0.53-0.75]), and CRP (AUC [95% CI]: 0.62 [0.53-0.71]). As shown in Table 3, IL-37 could improve the NIHSS score (AUC of the combined model 1, 0.75; 95% CI, 0.67–0.83; *P* < 0.001). The average AUC (standard error (SE)) for the NIHSS score and combined model 1 was 0.666 (0.047) and 0.751 (0.042), corresponding to a difference of 0.085 (0.005). Furthermore, IL-37 could improve the IL-6 and CRP (AUC of the combined model 2, 0.77; 95% CI, 0.70–0.85; *P* < 0.001). This improvement resulted in an average AUC (SE) of 0.641 (0.055) for IL-6, 0.624(0.046) for CRP, and 0.774 (0.040) for the combined model 2, corresponding to a difference of 0.133 (0.015) and 0.150 (0.006), respectively.

### 3.4. Subgroup Analysis

**Figure 6:**
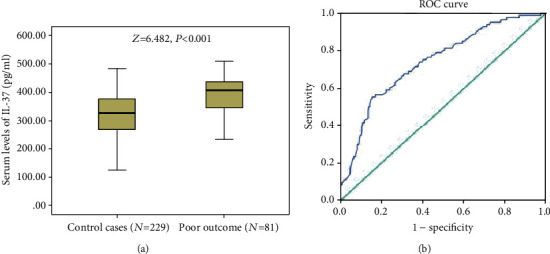
Serum levels of IL-37 and 3-month functional outcome. (a) Serum levels of IL-37 in stroke patients with poor outcome and good outcome. All data are medians and interquartile ranges (IQR). *P* values refer to Mann–Whitney *U* tests for differences between groups. (b) Receiver operating characteristic (ROC) curves were utilized to evaluate the accuracy of serum IL-37 levels to predict poor outcome.

At 3 months, 81 patients (26.1%) had a poor functional outcome (mRS, 3-6). The IL-37 serum levels in those patients were higher than in those patients with good outcome (407.4 pg/ml (341.7-436.7) vs. 327.4 pg/ml (IQR, 267.6-375.2); *Z* = 6.482, *P* < 0.001), Figure 6(a). In a multivariate logistic model adjusted for other factors, an IL-37 in the highest quartile was still associated with poor outcome (OR = 4.85; 95%CI = 3.11 − 8.22; *P* < 0.001). According to ROC analysis (Figure 6(b)), the cut-off value of IL-37 to predict poor outcome was suggested to be 401.5 pg/ml, with the AUC (95% CI) of 0.74 (0.68-0.80). The sensitivity and specificity at this concentration were 55.6% and 84.3%, respectively.

## 4. Discussion

The role of IL-37 in immune responses and innate inflammatory has still elusive [31], and the proinflammatory and anti-inflammatory role had been proposed [32]. This study set out with the aim of assessing the importance of IL-37 in ischemic stroke, and the results have shown that (1) serum IL-37 increased in ischemic stroke patients; (2) high IL-37 serum level was independently associated with stroke recurrence; (3) IL-37 was a novel indicator of recurrence events improving currently used factors; (4) high IL-37 level was associated with poor functional prognosis. Those data demonstrated a crucial role of this novel cytokine in poststroke pathology, suggesting it is a novel therapeutic strategy for patients with ischemic stroke. This study suggested future approaches to management should be emphasized for ischemic stroke with high IL-37 serum level (>405.3 pg/ml).

This finding is consistent with that of Zhang et al. [33], who found that elevated plasma levels of IL-37 were associated with poor 3-month outcomes (OR [95%CI] = 1.033 [1.015 − 10.56], *P* = 0.001) in ischemic stroke patients. In accordance with the present results, previous studies have demonstrated that serum IL-37 increased in patients after ischemic stroke [21, 34]. Similarly, another study reported that IL-37 plays an essential role in mediating poststroke inflammation with a significant increase in serum level in ischemic stroke patients [35]. However, another study showed that IL-37 expression might exert protective effects by reducing poststroke inflammation in the brain and periphery following ischemic stroke in IL-37tg mice [34].

A previous study confirmed that increased serum level of IL-37 was associated with a worse in-hospital outcome in patients with ST-segment elevation acute myocardial infarction [36]. Ji et al. [37] found that the high IL-37 plasma levels were associated with the onset of acute coronary syndrome (ACS), while another study revealed that autologous IL-37-treated tDCs was a useful therapeutic strategy for ACS [38]. Besides, IL-37 was elevated in patients with AF, and its expression was associated with AF subgroups [39]. Bello et al. [40] suggested that IL-37 was a natural modulator of inflammation via a feedback loop to suppress exuberant inflammatory immune reactions. Another study reported that IL-37 was a novel and potent proangiogenic cytokine with an essential role in pathophysiological settings in the mouse model of retinal vascular development [41]. These data showed that IL-37 represents newcomers to the spectrum of anti-inflammatory cytokines [42]. In this study, the data showed that serum IL-37 increased in patients after ischemic stroke and was associated with stroke recurrence, and poor outcomes might be a result caused by the consequences of unregulated cytokine activation, which eventually leads to immunopathological events.

It is very attractive to find early serum markers to predict stroke recurrence. Different variables, such as IL-6 [43], IL-33 [44], VCAM-1 [15], CRP [45], vitamin D [46], and copeptin [47], had been proposed as useful biomarkers for predicting stroke recurrence events in patients with ischemic stroke. Similarly, we also found that CRP and IL-6 were independent predictor biomarkers for stroke recurrence. Furthermore, age and atrial fibrillation had been reported as risk factors for stroke recurrence [48, 49], which our results had approved.

### 4.1. Strengths and Limitations

In this study, we investigated the IL-37 serum level in acute ischemic stroke and the value of predicting 3-month recurrence events and functional outcome in patients with ischemic stroke. There was no related literature report assessing the association between IL-37 serum level and stroke recurrence. Furthermore, in the multivariable model, some inflammatory markers such as CRP and IL-6 had been adjusted.

This study included limitations. First, observational studies with small samples (36 patients with stroke recurrence) were not determined to obtain useful results in a single center. Second, serum samples were only obtained on admission. We did not assess the continuous changes of serum biomarkers in stroke patients. A study showed that serum IL-37 levels postischemic stroke were 3–10 times elevated after acute ischemic stroke with an uptrend in the first few days [21]. Third, patients who died were excluded. Those patients might more likely suffer from a recurrent stroke. Selection bias might cause our data to be underestimated. Fourth, we tested serum levels of CRP, IL-6, and IL-37 and found that the prognostic accuracy of IL-37 was high compared to that of IL-6 and CRP. However, we did not test the levels of IL-1 and TNF-a, so we could not determine the association of those factors with IL-37 level and outcomes of ischemic stroke patients. Lastly, observational research could not lead to causal results. Whether IL-37 offered potential as a target for discovering novel immune-modulating anti-inflammatory therapies in stroke patients needs further verification.

## 5. Conclusion

Serum IL-37 increased in patients after ischemic stroke and was associated with stroke recurrence events and poor stroke outcomes. The underlying mechanisms of these associations need further elaboration. Large randomized controlled trials should be carried out to confirm whether IL-37 lowering treatment improves stroke prognosis.

## Figures and Tables

**Table 1:** Baseline characteristics of 310 stroke patients.

	Ischemic stroke patients
Age, medians (IQRs) (years)	66 (56-75)
Sex-female (*n* (%))	180 (58.1)
BMI, medians (IQRs) (kg/m^2^)	25.5 (23.9-26.8)
Vascular risk factors (*n* (%))	
Hypertension	199 (64.2)
Diabetes	101 (32.6)
Hypercholesterolemia	109 (35.2)
Atrial fibrillation	36 (11.6)
Cardiovascular diseases	59 (19.0)
TIA	28 (9.0)
Family history of stroke	21 (6.8)
Smoking	66 (21.3)
Drinking	59 (19.0)
Prestroke treatment (*n* (%))	
Antihypertensive	162 (52.3)
Hypoglycemic	81 (26.1)
Anticoagulants	26 (8.4)
Antiplatelet agents	77 (24.8)
Acute treatment (*n* (%))	
TPA-T	32 (10.3)
Mechanical thrombectomy	11 (3.5)
TPA-T and/or mechanical thrombectomy	38 (12.3)
Stroke etiology no. (%)	
Small-vessel occlusive	68 (21.9)
Large-vessel occlusive	90 (29.0)
Cardioembolic	106 (34.2)
Other	30 (9.7)
Unknown	16 (5.2)
NIHSS score at admission, medians (IQRs)	6 (3-11)
NIHSS < 6	141 (45.5)
Lesion volumes at admission (*N* = 175), median (IQR) (ml)	15 (5-31)
Laboratory findings at admission, medians (IQR)	
Il-37 (pg/ml)	344.1 (284.4-405.3)
Il-6 (ng/ml)	12.8 (9.2-16.9)
CRP (mg/l)	5.5 (2.4-9.3)
FSG (mmol/l)	5.81 (5.21-6.33)
Follow-up at 3-month	
mRS score, medians (IQR)	1 (0-2)
mRS > 2	81 (26.1)
Stroke recurrence (*n* (%))	36 (11.6)

IQR: interquartile range; BMI: body mass index; NIHSS: National Institutes of Health Stroke Scale; TPA-T: tissue plasminogen activator-treated; CRP: C-reactive protein; IL-37: interleukin-37; IL-6: interleukin-6; FSG: fasting serum glucose; mRS: modified Rankin scale.

**Table 2:** Multivariate analysis of predictors of stroke recurrence^†^.

Predictors	OR	95% CI	*P*
IL − 37 > 405.3 pg/ml^‡^	3.32	2.03-6.13	<0.001
NIHSS (per unit increase)	1.16	1.05-1.30	0.008
Stroke etiology (cardioembolic vs. other)	3.76	2.33-6.12	0.015
Atrial fibrillation (yes vs. no)	2.45	1.83-3.03	0.002
Acute treatment, TPA-T (yes vs. no)	0.32	0.25-0.40	<0.001
IL-6 (per unit increase)	1.21	1.11-1.30	0.003
CRP (per unit increase)	1.43	1.20-1.58	0.009

^†^The following variables were adjusted: age, sex, BMI, stroke etiology, vascular risk factors, prestroke and acute treatment, NIHSS score, and serum levels of IL-6, IL-37, CRP, and FSG. ^‡^The reference used for the analyses was the lowest three quartiles (Q1-Q3), corresponding to an IL − 37 level ≤ 405.3 pg/ml (Q4 vs. Q1-Q3). OR: odds ratio; CI: confidence interval; BMI: body mass index; NIHSS: National Institutes of Health Stroke Scale; TPA-T: tissue plasminogen activator-treated; CRP: C-reactive protein; IL-37; interleukin-37; IL-6: interleukin-6; FSG: fasting serum glucose.

**Table 3:** Area under the curve for selected predictors of stroke recurrence.

Test result variable(s)	Area	Std. error^†^	Asymptotic sig.^‡^	Asymptotic 95% CI	
				Lower bound	Upper bound
NIHSS	0.666	0.047	0.001	0.575	0.758
IL-37	0.733	0.042	<0.001	0.650	0.815
IL-6	0.641	0.055	0.006	0.533	0.748
CRP	0.624	0.046	0.016	0.534	0.714
Model 1(IL-37+ NHISS score)	0.751	0.042	<0.001	0.669	0.832
Model 2(IL-37 + IL-6 + CRP)	0.774	0.040	<0.001	0.696	0.853

^†^Under the nonparametric assumption. ^‡^Null hypothesis: true area = 0.5. CI: confidence interval; NIHSS: National Institutes of Health Stroke Scale; CRP: C-reactive protein; IL-37: interleukin-37; IL-6: interleukin-6.

## Data Availability

Please contact the corresponding author for data availability.

## References

[1] Zhou M., Wang H., Zeng X. (2019). Mortality, morbidity, and risk factors in China and its provinces, 1990-2017: a systematic analysis for the Global Burden of Disease Study 2017. *The Lancet*.

[2] Chao B.-H., Yan F., Hua Y. (2020). Stroke prevention and control system in China: CSPPC-Stroke Program. *International Journal of Stroke*.

[3] Xing L., Jing L., Tian Y. (2020). High prevalence of stroke and uncontrolled associated risk factors are major public health challenges in rural Northeast China: a population-based study. *International Journal of Stroke*.

[4] Wu S., Wu B., Liu M. (2019). Stroke in China: advances and challenges in epidemiology, prevention, and management. *The Lancet Neurology*.

[5] Feng Q., Yeung W. J. J., Wang Z., Zeng Y. (2019). Age of retirement and human capital in an aging China, 2015–2050. *European Journal of Population*.

[6] Sun H., Zou X., Liu L. (2013). Epidemiological factors of stroke: a survey of the current status in China. *Journal of Stroke*.

[7] Han J., Mao W., Ni J. (2020). Rate and determinants of recurrence at 1 year and 5 years after stroke in a low-income population in rural China. *Frontiers in Neurology*.

[8] Wang A., Wu L., Wang X. (2016). Effect of recurrent stroke on poor functional outcome in transient ischemic attack or minor stroke. *International Journal of Stroke*.

[9] Petty G. W., Brown R. D., Whisnant J. P., Sicks J. D., O'Fallon W. M., Wiebers D. O. (1998). Survival and recurrence after first cerebral infarction: a population-based study in Rochester, Minnesota, 1975 through 1989. *Neurology*.

[10] Jickling G. C., Sharp F. R. (2011). Blood biomarkers of ischemic stroke. *Neurotherapeutics*.

[11] Jin R., Yang G., Li G. (2010). Inflammatory mechanisms in ischemic stroke: role of inflammatory cells. *Journal of Leukocyte Biology*.

[12] Dziedzic T. (2015). Systemic inflammation as a therapeutic target in acute ischemic stroke. *Expert Review of Neurotherapeutics*.

[13] Nakase T., Yamazaki T., Ogura N., Suzuki A., Nagata K. (2008). The impact of inflammation on the pathogenesis and prognosis of ischemic stroke. *Journal of the Neurological Sciences*.

[14] Whiteley W., Jackson C., Lewis S. (2011). Association of circulating inflammatory markers with recurrent vascular events after stroke: a prospective cohort study. *Stroke*.

[15] Castillo J., Álvarez-Sabín J., Martínez-Vila E. (2009). Inflammation markers and prediction of post-stroke vascular disease recurrence: the MITICO study. *Journal of Neurology*.

[16] Di Napoli M., Papa F. (2002). Inflammation, hemostatic markers, and antithrombotic agents in relation to long-term risk of new cardiovascular events in first-ever ischemic stroke patients. *Stroke*.

[17] Wang X., Xu K., Chen S., Li Y., Li M. (2018). Role of interleukin-37 in inflammatory and autoimmune diseases. *Iranian Journal of Immunology*.

[18] McCurdy S., Liu C. A., Yap J., Boisvert W. A. (2019). Potential role of IL-37 in atherosclerosis. *Cytokine*.

[19] Yang Z., Kang L., Wang Y. (2019). Role of IL-37 in cardiovascular disease inflammation. *Canadian Journal of Cardiology*.

[20] Chai M., Zhang H. T., Zhou Y. J. (2017). Elevated IL-37 levels in the plasma of patients with severe coronary artery calcification. *Journal of Geriatric Cardiology: JGC*.

[21] Bushnaq S., Zafar A., Duraisamy K. (2017). Abstract TP224: interleukin-37 level is elevated in acute ischemic stroke. *Stroke*.

[22] Hatano S. (1976). Experience from a multicentre stroke register: a preliminary report. *Bulletin of the World Health Organization*.

[23] Brott T., Adams H. P., Olinger C. P. (1989). Measurements of acute cerebral infarction: a clinical examination scale. *Stroke*.

[24] Adams H. P., Bendixen B. H., Kappelle L. J. (1993). Classification of subtype of acute ischemic stroke. Definitions for use in a multicenter clinical trial. TOAST. Trial of org 10172 in acute stroke treatment. *Stroke*.

[25] Sims J. R., Gharai L. R., Schaefer P. W., Vangel M., Rosenthal E. S. (2009). ABC/2 for rapid clinical estimate of infarct, perfusion, and mismatch volumes. *Neurology*.

[26] Zhang Q., Ding H., Yan J. (2011). Plasma tissue kallikrein level is negatively associated with incident and recurrent stroke: a multicenter case–control study in China. *Annals of Neurology*.

[27] Huang H., Zheng T., Wang S., Wei L., Wang Q., Sun Z. (2016). Serum 25-hydroxyvitamin D predicts early recurrent stroke in ischemic stroke patients. *Nutrition, Metabolism and Cardiovascular Diseases*.

[28] Bonita R. B. R., Beaglehole R. (1988). Recovery of motor function after stroke. *Stroke*.

[29] Tu W. J., Zhao S. J., Xu D. J., Chen H. (2014). Serum 25-hydroxyvitamin D predicts the short-term outcomes of Chinese patients with acute ischaemic stroke. *Clinical Science*.

[30] Daubail B., Jacquin A., Guilland J. C. (2013). Serum 25-hydroxyvitamin D predicts severity and prognosis in stroke patients. *European Journal of Neurology*.

[31] Nold M. F., Nold-Petry C. A., Zepp J. A., Palmer B. E., Bufler P., Dinarello C. A. (2010). IL-37 is a fundamental inhibitor of innate immunity. *Nature Immunology*.

[32] Bai J., Li Y., Li M., Tan S., Wu D. (2020). IL-37 as a potential biotherapeutics of inflammatory diseases. *Current Drug Targets*.

[33] Zhang F., Zhu T., Li H. (2020). Plasma interleukin-37 is elevated in acute ischemic stroke patients and probably associated with 3-month functional prognosis. *Clinical Interventions in Aging*.

[34] Zhang S. R., Nold M. F., Tang S.-C. (2019). IL-37 increases in patients after ischemic stroke and protects from inflammatory brain injury, motor impairment and lung infection in mice. *Scientific Reports*.

[35] Zafar A., Ikram A., Jillella D. V. (2017). Measurement of elevated IL-37 levels in acute ischemic brain injury: a cross-sectional pilot study. *Cureus*.

[36] Liu K., Tang Q., Zhu X., Yang X. (2017). IL-37 increased in patients with acute coronary syndrome and associated with a worse clinical outcome after ST-segment elevation acute myocardial infarction. *Clinica Chimica Acta*.

[37] Ji Q., Zeng Q., Huang Y. (2014). Elevated plasma IL-37, IL-18, and IL-18BP concentrations in patients with acute coronary syndrome. *Mediators of Inflammation*.

[38] Mao X., Zhu R., Zhang F. (2019). IL-37 plays a beneficial role in patients with acute coronary syndrome. *Mediators of Inflammation*.

[39] Li W., Li S., Li X., Jiang S., Han B. (2017). Interleukin-37 elevation in patients with atrial fibrillation. *Clinical Cardiology*.

[40] Bello R., Chin V., Isnadi M. A. R. (2018). The role, involvement and function (s) of interleukin-35 and interleukin-37 in disease pathogenesis. *International Journal of Molecular Sciences*.

[41] Yang T., Lin Q., Zhao M. (2015). IL-37 is a novel proangiogenic factor of developmental and pathological angiogenesis. *Arteriosclerosis, Thrombosis, and Vascular Biology*.

[42] Banchereau J., Pascual V., O'garra A. (2012). From IL-2 to IL-37: the expanding spectrum of anti-inflammatory cytokines. *Nature Immunology*.

[43] Jia Q., Jiang F., Ma D., Li J., Wang F., Wang Z. (2020). Association between IL-6 and seizure recurrence in patients with the first post-ischemic stroke seizure. *Neuropsychiatric Disease and Treatment*.

[44] Li X. M., Wang X. Y., Feng X. W. (2019). Serum interleukin-33 as a novel marker for long-term prognosis and recurrence in acute ischemic stroke patients. *Brain and Behavior*.

[45] Li J., Zhao X., Meng X. (2016). High-sensitive C-reactive protein predicts recurrent stroke and poor functional outcome: subanalysis of the clopidogrel in high-risk patients with acute nondisabling cerebrovascular events trial. *Stroke*.

[46] Qiu H., Wang M., Mi D., Zhao J., Tu W., Liu Q. (2017). Vitamin D status and the risk of recurrent stroke and mortality in ischemic stroke patients: data from a 24-month follow-up study in China. *The Journal of Nutrition, Health & Aging*.

[47] Greisenegger S., Segal H. C., Burgess A. I., Poole D. L., Mehta Z., Rothwell P. M. (2015). Copeptin and long-term risk of recurrent vascular events after transient ischemic attack and ischemic stroke: population-based study. *Stroke*.

[48] Penado S., Cano M., Acha O., Hernández J. L., Riancho J. A. (2003). Atrial fibrillation as a risk factor for stroke recurrence. *The American Journal of Medicine*.

[49] Mohan K. M., Crichton S. L., Grieve A. P., Rudd A. G., Wolfe C. D. A., Heuschmann P. U. (2009). Frequency and predictors for the risk of stroke recurrence up to 10 years after stroke: the South London Stroke Register. *Journal of Neurology, Neurosurgery & Psychiatry*.

